# Bcl‐xL represents a therapeutic target in Philadelphia negative myeloproliferative neoplasms

**DOI:** 10.1111/jcmm.15730

**Published:** 2020-08-13

**Authors:** Jessica Petiti, Marco Lo Iacono, Valentina Rosso, Giacomo Andreani, Aleksandar Jovanovski, Marina Podestà, Dorela Lame, Marco De Gobbi, Carmen Fava, Giuseppe Saglio, Francesco Frassoni, Daniela Cilloni

**Affiliations:** ^1^ Department of Clinical and Biological Sciences University of Turin Turin Italy; ^2^ Department of Pediatric Hemato‐Oncology and Stem Cell and Cellular Therapy Laboratory Institute G. Gaslini Genova Italy

**Keywords:** ABT‐737, Bcl‐xL, myeloproliferative neoplasms, therapeutic target

## Abstract

Myeloproliferative neoplasms are divided into essential thrombocythemia (ET), polycythemia vera (PV) and primary myelofibrosis (PMF). Although ruxolitinib was proven to be effective in reducing symptoms, patients rarely achieve complete molecular remission. Therefore, it is relevant to identify new therapeutic targets to improve the clinical outcome of patients. Bcl‐xL protein, the long isoform encoded by alternative splicing of the *Bcl‐x* gene, acts as an anti‐apoptotic regulator. Our study investigated the role of Bcl‐xL as a marker of severity of MPN and the possibility to target Bcl‐xL in patients. 129 MPN patients and 21 healthy patients were enrolled in the study. We analysed *Bcl‐xL* expression in leucocytes and in enriched CD34+ and CD235a+ cells. Furthermore, ABT‐737, a Bcl‐xL inhibitor, was tested in HEL cells and in leucocytes from MPN patients. Bcl‐xL was found progressively over‐expressed in cells from ET, PV and PMF patients, independently by JAK2 mutational status. Moreover, our data indicated that the combination of ABT‐737 and ruxolitinib resulted in a significantly higher apoptotic rate than the individual drug. Our study suggests that Bcl‐xL plays an important role in MPN independently from JAK2 V617F mutation. Furthermore, data demonstrate that targeting simultaneously JAK2 and Bcl‐xL might represent an interesting new approach.

## 1 INTRODUCTION

Philadelphia chromosome‐negative (Ph‐) myeloproliferative neoplasms (MPN) are a heterogeneous group of diseases that includes essential thrombocythemia (ET), polycythemia vera (PV) and primary myelofibrosis (PMF).^1^ Different somatic alterations in *Janus Kinase 2* (*JAK2*), *Calreticulin* (*CALR*) and *Myeloproliferative Leukemia* (*MPL*) genes, that cause constitutive activation of the JAK2 signal, are considered as ‘drivers’ in MPN pathogenesis.^2^, ^3^, ^4^ For PV and ET patients with low risk of leukaemic transformation and fibrotic progression, the treatment has been focused on preventing cardiovascular events, such as thrombosis and haemorrhage.^5^ In contrast, PMF is an aggressive disease associated with an increased risk of leukaemic transformation. Ruxolitinib (Novartis, Basel, Switzerland), a JAK1/2 kinase inhibitor, was approved as first‐line therapy in PMF treatment, in myelofibrosis secondary to ET or PV (PET‐MF and PPV‐MF)^6^ and as second‐line therapy in PV patients who failed hydroxyurea.^7^ Although ruxolitinib was proven to be effective in reducing symptoms and spleen volume, patients rarely achieve complete molecular remission.^8^ Although allogeneic stem cell transplantation involves a high risk of graft‐related complications and mortality, it is nowadays the only curative treatment for myelofibrosis.^9^ For these reasons, it is clinically relevant to identify new molecular targets for innovative therapeutic strategies to improve the clinical outcome of MPN patients.

B‐cell lymphoma‐extra‐large^10^ (Bcl‐xL) protein is the long isoform encoded by alternative splicing of the *Bcl‐x* gene, a member of the Bcl‐2 family, with a mitochondrial localization in the outer membrane. Bcl‐xL forms heterodimers with BCL2 Associated X (BAX), BCL2 Antagonist/Killer (BAK), or B‐Cell CLL/Lymphoma 2 (BCL2) and acts as an anti‐apoptotic regulator, thus inhibits cytochrome C releasing into the cytosol.^11^ Its overexpression is confirmed in different types of cancers, such as breast, prostate and multiple myeloma, where it has been associated with a negative prognosis and poor overall survival.^12^, ^13^, ^14^ Bcl‐xL is also one of the most important factors responsible for the survival of erythroid progenitors that differentiate into mature erythrocytes.^15^ Despite the precise mechanism of Bcl‐xL regulation is still unclear, it is known that the *Bcl‐x* promoter contains consensus motifs for several transcription factors, among which the most important seem to be signal transducers and activator of transcription (STATs), nuclear factor kappa B (NF‐κB) and several members of the erythroblast transformation‐specific (ETS) family.^16^ In 2002, Kirito et al showed that *Bcl‐xL* expression in normal megakaryocytes is regulated by thrombopoietin through the activation of both JAK2 and Peptidase Inhibitor 3‐kinase (PI3K) signalling, inducing the binding of STAT5 and p50.^17^ Subsequently, Silva et al suggested that deregulated expression of *Bcl‐xL* may contribute to the erythropoietin‐independent survival of erythroid precursor in PV,^18^ and Tognon et al reported an increased *Bcl‐xL* expression in JAK2‐mutated ET and PMF leucocytes compared to control group.^19^ In 2009, a study of a small cohort of PV *JAK2* V617F mutated patients highlighted that 25% of erythroid precursors express elevated *Bcl‐xL* levels and its inhibition with ABT‐737 interferes with erythroid colonies formation.^20^

ABT‐737 (AbbVie, North Chicago, IL, USA) is a small Bcl‐2 homology 3 (BH3) mimetic inhibitor of the Bcl‐2 family, with high binding affinity for Bcl‐xL. Previous data identified its pro‐apoptotic and antineoplastic activity in several hematopoietic and solid tumour cell lines and xenograft models.^21^, ^22^, ^23^ ABT‐737 binds the hydrophobic groove of Bcl‐xL and restores apoptotic processes, via activation of BAK/BAX.

In this study, we aimed to investigate the expression of Bcl‐xL in different cell populations of MPN patients, emphasizing its role as a marker for the clinical severity among the MPN subgroups. The co‐inhibition of the JAK2 oncogenic pathway in two points, upstream (JAK2) and in the effector phase (Bcl‐xL), has proved to be extremely effective in the treatment of TEL‐JAK2‐positive T‐cell acute lymphoblastic leukaemia, both in cell lines and in mice models.^24^ Herein, we demonstrate that concurrent inhibition of these two targets boosts the apoptotic cell response in MPN patients and in HEL cells. These results pave the way to develop future therapeutic strategies for MPN cells giving relevant clues for the development of novel treatment approaches for MPN patients.

## 2 MATERIALS AND METHODS

### 2.1 Cells culture conditions

HEL cell line, human erythroleukaemia cells homozygous for JAK2 V617F mutation, was purchased from American Type Culture Collection (ATCC, Manassas, USA). HEL cells were grown in RPMI 1640 medium (EuroClone, Milan, Italy) supplemented with 10% inactivated foetal bovine serum (FBS) (Sigma‐Aldrich, St. Louis, MO, USA), 200 nmol\L Glutamine (EuroClone, Milan, Italy) and 0.1% penicillin/streptomycin at a temperature of 37°C with 5% CO_2_.

### 2.2 Cohort of patients and CD34/CD235a cell enrichment

After signing written informed consent, 129 MPN patients (46 ET, 47 PV and 36 MF) and 21 healthy patients were enrolled in the study. Sixty patients were *JAK2* wild‐type and 69 *JAK2* V617F. Peripheral blood (PB) leucocytes were isolated by Buffy Coat. Hematopoietic and erythroid progenitor cells were enriched by magnetic cell sorting (MACS) using, respectively, CD34 and CD235a (Glycophorin A) MicroBead Kits (Miltenyi Biotec, Bergisch Gladbach, Germany), according to the manufacturer's protocol. After enrichment, the purity of CD34+ and CD235a+ was evaluated by FACS and samples with a purity lower than 95% were excluded from further analysis.

### 2.3 RNA extraction and qRT‐PCR analysis

Total RNA was extracted using TRIzol Reagent (Ambion, Thermo Fisher Scientific, Waltham, MA, USA) following the manufacturer's instructions and was reverse transcribed to complementary DNA as previously described.^25^ The expression level of *Bcl*‐*xL* (Assays‐on‐Demand™ Hs01070110_m1, Thermo Fisher Scientific, Waltham, MA, USA) was evaluated with TaqMan technology (TaqMan Universal Master Mix, Thermo Fisher Scientific, Waltham, MA, USA), by using the C1000 Thermal Cycler CFX96 Real‐Time System (Bio‐Rad, Hercules, CA, USA). Gene expression was normalized with respect to *Abelson 1* (*ABL1)* (Assays‐on‐Demand™ Hs00245445_m1, Thermo Fisher Scientific, Waltham, MA, USA). Results were analysed by Bio‐Rad CFX Manager 3.1 software (Bio‐Rad, Hercules, CA, USA) and indicated as ‐ΔCt [‐(Ct*_Bcl‐xL_*‐Ct*_ABL1_*)].

### 2.4 Immunofluorescence and image analysis

Cytospin was used to immobilize CD34+ and CD235a+ cells from MPN patients onto glass slides. Immunofluorescence analysis was performed as previously described.^26^ Briefly, cells were fixed with 4% paraformaldehyde permeabilized with 0.1% Triton x‐100. After saturation, cells were incubated with Bcl‐xL primary antibody (H‐5 sc‐8392, Santa Cruz Biotechnology, Dallas, TX, USA) with a dilution of 1:100 in blocking solution for 2 hours (hrs) at room temperature (RT). Afterwards, slides were incubated with Alexa 488‐conjugated secondary antibody (A32723, ThermoFisher Scientific, Waltham, MA, USA) diluted 1:1000 in 1x Phosphate‐buffered saline (PBS) for 1 hr at RT and with 1 μg/mL 4′,6‐diamidino‐2‐phenylindole (DAPI) for 5 min in the dark. Cells were mounted with Mowiol and fluorescent signals analysed with a confocal scanning microscope (LSM 5110; Carl Zeiss MicroImaging Inc, Oberkochen, Germany), using a 63 × objective. For each sample, at least 100 cells were acquired and fluorescence intensity was measured in arbitrary units (a.u.) by the Image J program (https://imagej.nih.gov/ij/).

### 2.5 Cells treatment

HEL cells were incubated with different concentrations of ABT‐737 (AbbVie, Chicago, IL, USA) and ruxolitinib (Novartis, Basel, Switzerland) for 24 hrs (stock solutions in 10 mmol\L in Dimethyl sulfoxide (DMSO)). Leucocytes isolated from MPN patients were incubated with 2.5 µmol\L of ABT‐737 and 5 µmol\L of ruxolitinib in Iscove's Modified Dulbecco's Media (IMDM) (EuroClone, Milan, Italy) supplemented with 20% inactivated FBS for 24 hrs. The concentrations of ABT‐737 and ruxolitinib used to treat cells were chosen based on the results of the proliferation experiments (IC50) and the literature. After incubation, total RNA and proteins were extracted and proliferation and apoptosis were evaluated.

### 2.6 Proliferation and apoptosis assays

Proliferation was evaluated by XTT assay (Cell Proliferation Kit II (XTT), Sigma‐Aldrich, Thermo Fisher, Waltham, MA, USA), according to the manufacturer's instructions. Experiments were performed in quadruplicate. HEL cells were cultured for 24 hrs testing different drug concentrations (range: 0‐20 µmol\L for both drugs). The combined effect of the drugs was tested by mixing 1:1 the doses used for every single treatment. The half‐maximal inhibitory concentration at 24 hrs (IC_50_) for ABT‐737 and ruxolitinib, alone and in combination, was calculated by evaluating the viability data with Sigmoidal Dose‐Response analysis using GraphPad Prism 7.00 software. The combination index (CI) was calculated using the mutually non‐exclusive formula as previously described.^27^ For the analysis, the synergism between drugs was defined when CI value was <1.

Apoptosis was evaluated by FACS measuring annexin/PE staining (Annexin V‐FITC Apoptosis Detection Kit, Immunostep, Salamanca, Spain) in HEL cells and leucocytes from MPN patients, following the manufacturer's protocol. At least 100,000 events were acquired for each condition. Data analysis was performed using BD CellQuest software (BD Biosciences, San Jose, CA, USA).

### 2.7 Protein extraction and immunoblotting

HEL cells and leucocytes from MPN patients were used for immunoblotting assays. Total proteins were isolated with radioimmunoprecipitation assay (RIPA) buffer and quantified as previously described.^28^ 70 µg of each cell lysate was loaded, resolved through SDS‐PAGE 6%‐12% gel and electroblotted onto 0.2 µm PVDF membranes (GE Healthcare, Chicago, IL, USA). After blocking with 5% non‐fat milk (Sigma‐Aldrich, Thermo Fisher Scientific, Waltham, MA, USA), membranes were incubated 2 hrs at RT with Bcl‐xL (H‐5 sc‐8392) or overnight at 4°C with Phospho‐Jak2 (Tyr) (3771, Cell Signaling Technology, Danvers, MA, USA), JAK2 (C‐14 sc‐34479), GAPDH (A‐3 sc‐137179) and β‐Actin (C4 sc‐47778) [Santa Cruz Biotechnology, Dallas, TX, USA] primary antibodies with a dilution of 1:2000. Then, membranes were incubated with specific horseradish peroxidase (HRP)‐conjugated secondary antibodies: anti‐rabbit (sc‐2357), antimouse (sc‐2005) and anti‐goat (sc‐2354) [Santa Cruz Biotechnology, Dallas, TX, USA] in a dilution of 1:7000 for 1 hour at RT. Immunoreactive bands were visualized by Clarity Western ECL Substrate (Bio‐Rad, Hercules, CA, USA) and acquired by the Image Lab program (Bio‐Rad, Hercules, CA, USA).

### 2.8 Statistical analysis

Statistical analyses were performed using GraphPad Prism 7.00 software. Differential Bcl‐xL protein and transcript expression between healthy donors and MPN were evaluated using the two‐tailed *t* test. Two‐tailed paired *t* test was used to estimate differential Bcl‐xL mRNA expression and apoptosis rate between untreated and treated samples (HEL cell line and MPN leucocytes). All the analysis with a *P *< 0.05 were indicated as statistically significant (*P ≤ 0.05; **P ≤ 0.01; ***P ≤ 0.001; ****P ≤ 0.0001).

## 3 RESULTS

### 3.1 Mature leucocytes, hematopoietic progenitors and erythroblasts of MPN patients over‐express Bcl‐xL differently and independently from JAK2 mutational status

**FIGURE 1:**
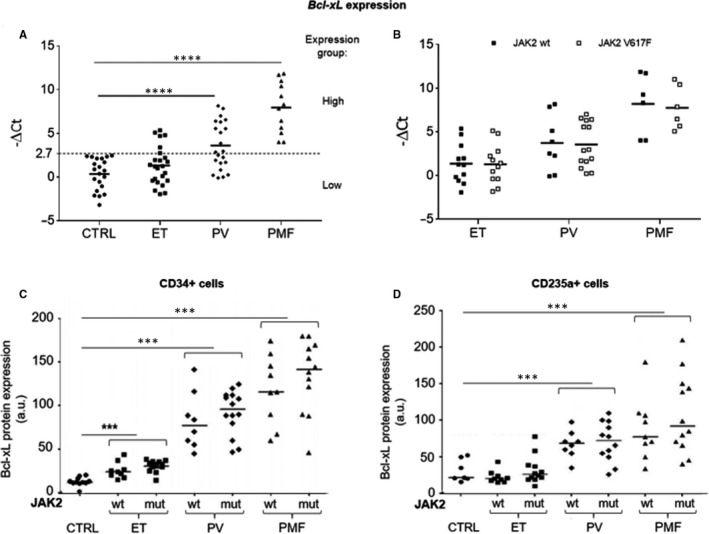
A, *Bcl*‐*xL* mRNA expression in mature leucocytes from healthy donors (ctrl) and MPN patients (ET, essential thrombocythemia; PV, polycythemia vera; and PMF, myelofibrosis). Primary samples were divided into two groups based on high or low *Bcl‐xL* expression by dichotomizing for the average value. B, *Bcl*‐*xL* mRNA expression in leucocytes from ET, PV and PMF, classified as *JAK2* wild‐type and mutated. C, Quantification of Bcl‐xL protein after immunofluorescence analysis in CD34 + cells (hematopoietic progenitor cells) from healthy donors and *JAK2* wild‐type (wt) and mutated (mut) MPN patients. D, Quantification of Bcl‐xL expression after immunofluorescence analysis in CD235 + cells (erythroblasts) from healthy donors and *JAK2* wt and mut MPN patients. Fluorescence intensity was measured in arbitrary units (a.u.)

To ascertain the role of the anti‐apoptotic *Bcl‐xL*, the long isoform of *Bcl‐x,* in Ph‐ MPN, we first evaluated its mRNA expression in mature leucocytes from healthy patients and from ET, PV and PMF patients. We observed a peculiar behaviour where *Bcl*‐*xL* expression increases from ET to PV to PMF, with a significative over‐expression in PV and MF groups (*P* < .001 for both) compared to the control group (Figure 1A). Then, dichotomizing all the values according to the average expression in high and low expression groups, we observed a difference in the distribution among Ph‐ MPN patients in accordance with the clinical severity of the disease. Indeed, in the high expression group, we found none of the healthy patients, 25% of the ET patients, 55% of the PV, and all the PMF patients (Figure 1A). Furthermore, this behaviour was not influenced by the JAK2 V617F mutation. Indeed, as showed in Figure 1B, no differences in *Bcl*‐*xL* mRNA expression were observed when we stratified the patients according to JAK2 V617F mutation.

Considering the crucial role that Bcl‐xL plays in the regulation of proliferation/apoptosis of progenitor cells and erythroblasts, we decided to investigate the Bcl‐xL protein level. Hence, we quantified the protein amount by immunofluorescence assay in Ph‐ MPN cell populations enriched for CD34 and for CD235a positivity.

Coherently with the mRNA results, we observed that PMF cells showed the highest expression of Bcl‐xL protein (*P* < 0.01, compared with control), followed by PV (*P* < 0.01, compared with control) and ET (Figure 1C,D). The only difference identified between CD34+ and CD235a+ populations was the high significant expression of Bcl‐xL protein in all evaluated classes of CD34+ cells, compared with the control cells (*P* < 0.01, for each subgroup). In addition, also at the protein level, the presence of the JAK2 V617F mutation did not affect Bcl‐xL expression. Altogether, these results suggest that both Bcl‐xL mRNA and protein were over‐expressed in Ph‐ MPN independently from the JAK2 mutational status and correlated with the clinical severity of the diseases.

### 3.2 In vitro co‐inhibition of JAK2 and Bcl‐xL has a synergic effect and induces apoptosis in HEL cell line

**FIGURE 2:**
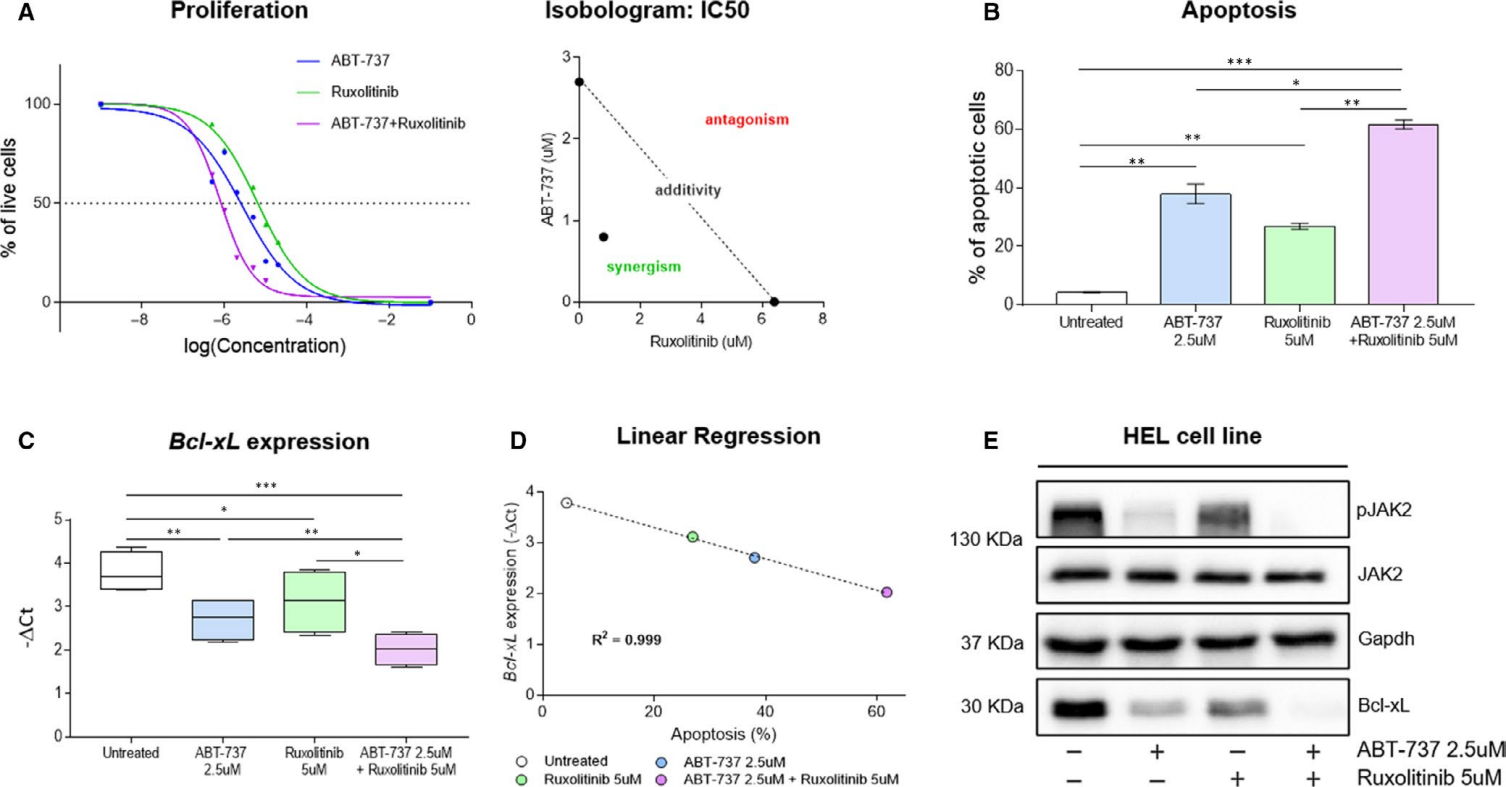
Effects of JAK2 and Bcl‐xL inhibition in HEL cells. A, Left: Sigmoidal Dose‐Response curves in HEL cells treated with different concentrations (range: 0‐20 µmol\L) of ABT‐737, ruxolitinib, and ABT‐737 + ruxolitinib for 24 hrs. The experiment was performed in quadruplicate. Right: isobologram for IC50. B, Annexin V apoptosis assay was performed to estimate the apoptosis rate in HEL cells after treatment with 2.5 µmol\L ABT‐737, 5 µmol\L ruxolitinib and ABT‐737 + ruxolitinib for 24 hrs. The bar graph showed the percentage of apoptotic cells. C, *Bcl‐xL* mRNA expression was evaluated in untreated HEL cells and after treatment with 2.5 µmol\L ABT‐737, 5 µmol\L ruxolitinib and ABT‐737 + ruxolitinib for 24 hrs. D, Correlation between apoptosis rate and *Bcl‐xL* modulation. Linear regression analyses were performed plotting *Bcl‐xL* mRNA expression (average value for each condition, *y*‐axis) and the percentage of apoptotic cells (average value for each condition, *x*‐axis) in HEL cells treated with 2.5 µmol\L ABT‐737, 5 µmol\L ruxolitinib and drugs combination for 24 hrs. E, JAK2 phosphorylation status and Bcl‐xL protein expression were evaluated by SDS‐PAGE in untreated HEL cells and after treatment by single or combined administration of 2.5 µmol\L ABT‐737 and 5 µmol\L ruxolitinib for 24 hrs. The Western blot assays, shown here, represent an example of at least 4 independent experiments

Having observed that the over‐expression of Bcl‐xL did not depend exclusively on the JAK2 signal, we have been suggested that the co‐inhibition of both these targets could have shown a synergistic effect in Ph‐MPN cells. To validate this hypothesis, we treated HEL cells with different concentrations (range: 0‐20 µmol\L) of ruxolitinib and ABT‐737, alone and in combination (A + R), for 24 hrs. Proliferation assay identified an IC50 of 2.7, 6.4 and 0.8 µmol\L for ABT‐737, ruxolitinib, and A + R, respectively. The CI of the two compounds was 0.44, indicating a strong synergistic inhibitory effect, as shown by the Sigmoidal Dose‐Response curves and the isobologram for IC50 (Figure 2A). Furthermore, we investigated the anti‐apoptotic effect of drugs. Our results indicated that the tested drugs significantly induced a high rate of apoptosis in HEL cells, either alone or in combination. Particularly, ABT‐737 and ruxolitinib showed a significant increase of apoptotic fraction of about 33.7% (*P* < 0.01) and 22.6% (*P* < 0.01), respectively, compared to the untreated cells. The combined treatment increased the apoptotic fraction of about 57.4% (*P* < 0.001) compared to control cells, resulting also in a significantly higher apoptotic rate over the single drug treatments (*P* < 0.05 vs. ABT‐737 and *P* < 0.01 vs. ruxolitinib). Thus, our results reveal a boosted anti‐apoptotic effect when both JAK2 and Bcl‐xL signals were inhibited (Figure 2B). In addition, we investigated how drugs affected *Bcl‐xL* mRNA modulation in HEL cells. Our data showed a significant down‐modulation of *Bcl‐xL* when cells were exposed to both ABT‐737 (*P* < 0.01), ruxolitinib (*P* < 0.05) and A + R (*P* < 0.001), compared to untreated cells. Interesting, the combination of drugs resulted in a significant reduction of *Bcl‐xL* expression also when compared to the drugs alone (*P* < .01 compared to ABT‐737 and *P* < .05 compared to ruxolitinib) (Figure 2C). These data seem to demonstrate that the Bcl‐xL expression or mRNA stabilization is dependent on JAK2‐STAT signalling, as suggested by Bcl‐xL down‐modulation mediated by ruxolitinib, that principally targets the JAK2‐STAT pathway. In addition, we observed a strong inverse correlation between *Bcl‐xL* modulation and apoptosis rate (Figure 2D; Pearson *r* = −0.99, *P* < 0.001), suggesting that apoptosis in HEL cells might be strictly dependent on *Bcl‐xL* transcriptional levels. Finally, we evaluated JAK2 phosphorylation status and Bcl‐xL protein levels in HEL cells. We found that Bcl‐xL mRNA and protein expression showed comparable patterns. Indeed, the Bcl‐xL and phospho‐JAK2 proteins levels slightly decreased after ruxolitinib treatment, and they are reduced a bit more with ABT‐737. Eventually, their levels appeared even further inhibited by the co‐treatment (Figure 2E). Also in this case, the crosstalk between the two targets was evident, as suggested by the presence of less phospho‐JAK2 protein when Bcl‐xL signalling was blocked by ABT‐737.

### 3.3 In vitro co‐inhibition of JAK2 and Bcl‐xL induces apoptosis in leucocytes from PV and PMF patients

**FIGURE 3:**
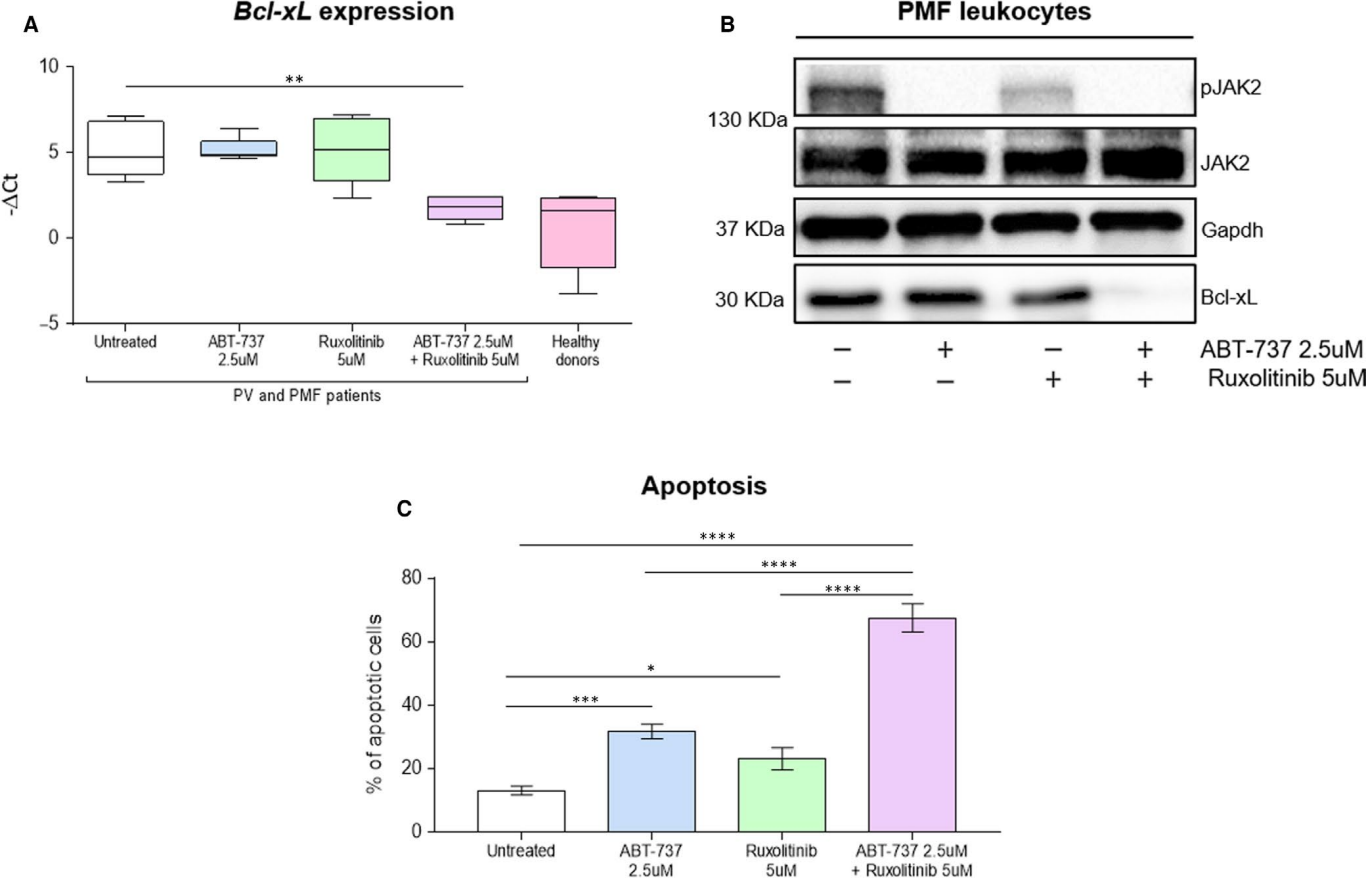
Effects of JAK2 and Bcl‐xL inhibition in leucocytes from PV and PMF patients, both JAK2 mutated and wild‐type. A, *Bcl‐xL* mRNA expression was evaluated in untreated cells from PV and PMF patients and after treatment with 2.5 µmol\L ABT‐737, 5 µmol\L ruxolitinib and ABT‐737 + ruxolitinib for 24 hrs. In addition, *Bcl‐xL* levels in healthy patients were reported. B, JAK2 phosphorylation status and Bcl‐xL protein expression were evaluated by SDS‐PAGE in untreated cells from PV and PMF patients and after treatment with single or combined drugs at the concentration of 2.5 µmol\L for ABT‐737 and 5 µmol\L for ruxolitinib for 24 hrs. The Western blot assays shown here in PMF leucocytes is representative of at least 4 independent experiments. C, Annexin V apoptosis assay was performed to estimate the apoptosis rate in cells from PV and PMF patients after treatment with 2.5 µmol\L ABT‐737, 5 µmol\L ruxolitinib and ABT‐737 + ruxolitinib for 24 hrs. The bar graph showed the percentage of apoptotic cells. The Western blot assays, shown here, represent an example of at least 4 independent experiments

Given the interesting results obtained in the HEL cell line, we decided to investigate the effect of ABT‐737 and ruxolitinib also in primary leucocytes from PV and PMF patients, both JAK2 mutated and wild‐type. ET patients were excluded from this analysis because they did not show a significative difference in Bcl‐xL expression compared to controls (Figure 1A). We treated in vitro leucocytes from PV and PMF patients with sub‐lethal doses close to IC_50_: 5 µmol\L ruxolitinib and 2.5 µmol\L ABT‐737, alone and in combination. First, we evaluated *Bcl‐xL* mRNA expression by qRT‐PCR. Different from what was observed in the cell line, in this case, we found that *Bcl‐xL* levels were not affected by the administration of ABT‐737 or ruxolitinib alone, but it was significantly down‐modulated (*P* < 0.01) only when drugs were used together (Figure 3A). This suggests a strong inhibitory effect only when both JAK2 and Bcl‐xL were targeted. Interestingly, *Bcl‐xL* expression in PV and PMF leucocytes after the treatment with A + R returned to the levels measured in healthy donors (Figure 3A).

In the analysis carried out in patients, we confirmed that Bcl‐xL modulation was affected by the combination of the drugs also at the protein level. Indeed, our data showed that Bcl‐xL expression was slightly inhibited when cells were treated only with ruxolitinib, no variation was observed after treatment with ABT‐737 only, while protein was strongly suppressed when the two drugs were used together (Figure 3B). We also investigated the phosphorylation status of JAK2, and we observed that the results in patients are similar to what found in cell line: phospho‐JAK2 levels were slightly decreased after ruxolitinib treatment, while were significantly reduced by ABT‐737 alone or in combination with ruxolitinib (Figure 3B). The difference in mRNA expression and proteins phosphorylation observed between cell lines and specimens from patients could be easily justified by the high heterogeneity of patients compared to a clonal cell line. To note that also in these cases, for both analyses and in accordance with in vitro results, we observed a maximal effect with drugs combination. Furthermore, we evaluated the apoptotic effects of the two drugs in leucocytes from PV and PMF patients and we obtained comparable data and even more evident than cell line experiments: the apoptotic fraction was increased of 10% (*P* < 0.05) with ruxolitinib, 18.6% (*P* < 0.001) with ABT‐737 and 54.4% (*P* < 0.0001) after A + R treatment, compared to untreated cells. The apoptotic rate was also significantly higher after treatment with the combination of drugs when compared with drugs alone (*P* < .0001) (Figure 3C), indicating a boosted anti‐apoptotic effect: about double of single drugs sum when both JAK2 and Bcl‐xL pathways were inhibited.

## 4 DISCUSSION

Ph‐ MPN are clonal disorders of the myeloid lineage that include three different subgroups: ET, PV and PMF. Progression to acute leukaemia can occur in any subgroup of MPN but is more frequent in PMF than PV and ET.^29^ At the same time, PV and ET can experience disease transformation into PMF and this outcome is more likely to occur in PV patients.^30^ In recent years, several molecular alterations have been identified, helping to better define the biological basis of these diseases. The most frequent alterations involve the *JAK2*, *CALR* and *MPL* genes, which imply the deregulated activation of the JAK2‐STAT pathway.^2^, ^3^, ^31^ Despite the discovery of these mutations, the clinical differences between ET, PV and PMF are still completely unexplained. This study aimed to identify additional targets that may characterize MPN. Experimental evidence indicates that Bcl‐xL, the long anti‐apoptotic isoform of the *Bcl‐x* gene, plays as a key regulator of apoptosis in myeloid cells, especially in the erythroid lineage.^32^ We have been suggested that Bcl‐xL could have a critical role in defining and characterizing the three subclasses of Ph‐ MPN. Our analysis highlight that both Bcl‐xL mRNA and protein levels are up‐regulated in total leucocyte and in erythroblasts of PV and PMF and in hematopoietic progenitors of patients from all MPN subgroups compared to healthy donors, confirming and implementing published data.^18^, ^19^ Indeed, although previously published studies have focused on the relationship between *JAK2* V617F mutation and *Bcl‐xL* expression, it is interesting to note that our data show that the mutational status of the *JAK2* gene does not directly affect the Bcl‐xL expression. This result suggests that in MPN the Bcl‐xL gene and protein expression may be correlated with the deregulation of the JAK2‐STAT pathway rather than with the JAK2 mutations. Furthermore, we found a peculiar behaviour of *Bcl‐xL* expression levels that increase from ET to PV with a maximum in PMF. This result is particularly attractive because *Bcl‐xL* appeared to be the first marker whose modulation is correlated with the clinical severity of MPN subgroups and risk of leukaemic evolution. Indeed, *Bcl‐xL* increases from ET, the less severe condition, to PV and finally to PMF, the form with the highest risk of leukaemia evolution.^33^, ^34^ It is plausible to assume that the overexpression of this important anti‐apoptotic protein could extend the life of the cells, favouring the accumulation of mutations and promoting the disease progression.

Although ruxolitinib blocks the JAK2 pathway, thus inhibiting STAT proteins phosphorylation, it only induces a weak pro‐apoptotic effect on HEL cells.^35^ Our results confirmed this observation and, at the same time, showed that ruxolitinib has only a weak effect on Bcl‐xL down‐modulation. This implies that *Bcl‐xL* expression in HEL cells is only partially dependent on JAK2 activation, supporting the hypothesis of the advantages to pharmacologically block both the targets.

We also found that *Bcl‐xL* transcriptional levels strictly affect the apoptosis in HEL cells, proving the critical role exert by Bcl‐xL in the survival of MPN leukocites.^15^ We also confirmed that ruxolitinib alone slightly inhibits JAK2 phosphorylation.^36^ Surprisingly, we found that the use of ABT‐737, alone or in co‐treatment, can suppress the JAK2 pathway directly upstream. This could be explained by the activation of BAX mediated by the ABT‐737 previously described by Vaux et al^37^ Indeed, as already observed in breast cancer by Lin et al,^38^ BAX activation may down‐regulate upstream the JAK2 signal. In addition, our results suggested that the combined use of ruxolitinib together with a compound capable of inhibiting the anti‐apoptotic Bcl‐xL isoform, such as the ABT‐737, could increase the power of treatment. In particular, in vitro experiments in HEL cell line highlighted a strong synergism between ABT‐737 and ruxolitinib, which was reflected in a considerable increase in apoptotic rate and, simultaneously, in a strong down‐regulation of Bcl‐xL levels, according to results previously obtained in TEL‐JAK2 T‐cell acute lymphoblastic leukaemia.^24^ Our preliminary data regarding apoptosis and JAK2 phosphorylation in leucocytes from patients support this hypothesis also in PV and PMF. Indeed, we found that the drugs combination treatment was able to down‐modulate Bcl‐xL expression, returning it to the levels observed in healthy donors. Altogether, our results emphasize the potential therapeutic effect because of the simultaneous inhibition of both targets.

In conclusion, this study identifies for the first time Bcl‐xL as a potential marker of clinical severity and risk of progression of MPN patients, independently from JAK2 mutational status. Furthermore, our data provided evidence that PV and PMF, either JAK2 mutated or wild‐type, could benefit from the concomitant inhibition of both JAK2 and Bcl‐xL signalling, indicating Bcl‐xL as a putative target for future therapeutic approaches.

## CONFLICT OF INTEREST

The authors declare that they have no competing interests.

## AUTHORS' CONTRIBUTION

JP and MLI: Experiment design; experiment; manuscript writing; and manuscript revision. VR, AJ, MP and DL: Experiments. GA, CF and MDG: Clinical data and data analysis. GS: Study supervision and final approval. FF and DC: Study design; manuscript writing; manuscript revision; and final approval. All authors: Manuscript reading and final manuscript approval.

## Data Availability

Data sharing not applicable to this article as no data sets were generated or analysed during the current study.
